# Once- versus twice-weekly carfilzomib in relapsed and refractory multiple myeloma by select patient characteristics: phase 3 A.R.R.O.W. study subgroup analysis

**DOI:** 10.1038/s41408-020-0300-y

**Published:** 2020-03-09

**Authors:** Meletios A. Dimopoulos, Ruben Niesvizky, Katja Weisel, David S. Siegel, Roman Hajek, María-Victoria Mateos, Michele Cavo, Mei Huang, Anita Zahlten-Kumeli, Philippe Moreau

**Affiliations:** 1 National and Kapodistrian University of Athens, Athens, Greece; 2 Center for Myeloma, New York Presbyterian Hospital-Weill Cornell Medical Center, New York, NY USA; 3 University Medical Center Hamburg-Eppendorf, Hamburg, Germany; 4 University Hospital of Tuebingen, Tuebingen, Germany; 5 John Theurer Cancer Center at Hackensack University Medical Center, Hackensack, NJ USA; 6 Department of Hematooncology, University Hospital Ostrava and Faculty of Medicine, University of Ostrava, Ostrava, Czech Republic; 7 Hematology Service, University Hospital, Salamanca, Spain; 8 “Seràgnoli” Institute of Hematology and Medical Oncology, Bologna University School of Medicine, Bologna, Italy; 9 Amgen, Inc., Thousand Oaks, CA USA; 10 Hematology Department, University Hospital Hotel-Dieu, Nantes, France

**Keywords:** Cancer, Medical research

## Abstract

The phase 3 A.R.R.O.W. study demonstrated that treatment with once-weekly carfilzomib (70 mg/m^2^) and dexamethasone (once-weekly Kd70 mg/m^2^) improved progression-free survival compared with twice-weekly carfilzomib (27 mg/m^2^) and dexamethasone (twice-weekly Kd27 mg/m^2^) in patients with relapsed and refractory multiple myeloma (RRMM; median, 11.2 versus 7.6 months; hazard ratio [HR] = 0.69; 95% confidence interval, 0.54–0.88; *P* = 0.0029). Once-weekly dosing also improved response rates and depth of response. We performed a subgroup analysis from A.R.R.O.W. according to age (<65, 65–74, or ≥75 years), renal function (creatinine clearance <50, ≥50–<80, or ≥80 mL/min), number of prior therapies (2 or 3), and bortezomib-refractory status (yes or no). Compared with twice-weekly Kd27 mg/m^2^, once-weekly Kd70 mg/m^2^ reduced the risk of progression or death (HR = 0.60–0.85) and increased overall response rates in nearly all the examined subgroups, consistent with reports in the overall A.R.R.O.W. population. The safety profiles of once-weekly Kd70 mg/m^2^ across subgroups were also generally consistent with those in the overall population. Findings from this subgroup analysis generally demonstrate a favorable benefit–risk profile of once-weekly Kd70 mg/m^2^, further supporting once-weekly carfilzomib dosing as an appropriate treatment option for patients with RRMM, regardless of baseline patient and disease characteristics.

## Introduction

Multiple myeloma (MM) is the third most common hematologic malignancy worldwide, characterized by excessive proliferation of monoclonal plasma cells^1,2^. The development of novel anti-MM agents has expanded treatment options for MM patients and improved outcomes^3^. Despite recent treatment advances, MM remains incurable, with most patients relapsing and developing treatment-refractory disease^1^. Relapsed and refractory MM (RRMM) represents a challenging disease to treat, given the heterogeneity of the disease and patient population^3–5^.

Importantly, advanced age, Eastern Cooperative Oncology Group performance status (ECOG PS), International Staging System (ISS), renal impairment, exposure to multiple lines of therapy, refractoriness to treatment, and the presence of high-risk cytogenetics have been associated with poor prognosis and shorter survival in patients with MM (including RRMM)^6–10^. Therefore, there is a continued need to identify safe and efficacious and ultimately convenient treatments across the heterogeneous RRMM patient population. For treatments with demonstrated safety and efficacy, convenience represents an important factor for optimizing adherence and patient quality of life^11–13^.

Carfilzomib (K) is a second-generation proteasome inhibitor (PI) with selective, irreversible, robust, and well-tolerated activity in MM, both as single agent^14^ and in combination with dexamethasone (Kd) or lenalidomide plus dexamethasone (KRd) when administered as a twice-weekly infusion in patients with RRMM^15^. Efficacy and safety of twice-weekly carfilzomib-based therapies were previously demonstrated in the phase 3 ASPIRE and ENDEAVOR trials^16–19^. Importantly, the treatment effect of carfilzomib was confirmed across several patient subgroups^20–26^. The favorable benefit–risk profile of twice-weekly carfilzomib-based therapies versus standards of care (SOCs) provided an opportunity to consider the value of more convenient once-weekly carfilzomib therapy for patients with RRMM.

The randomized phase 3 A.R.R.O.W. trial (NCT02412878) was designed to evaluate a once-weekly carfilzomib dosing schedule compared with twice-weekly administration in patients with RRMM^27^. Patients were randomly assigned to receive once-weekly carfilzomib (70 mg/m^2^) with dexamethasone (once-weekly Kd70-mg/m^2^ arm) or twice-weekly carfilzomib (27 mg/m^2^) with dexamethasone (twice-weekly Kd27-mg/m^2^ arm). In the prespecified interim analysis, progression-free survival (PFS) and overall response rate (ORR) were improved in the once-weekly Kd70-mg/m^2^ arm versus the twice-weekly Kd27-mg/m^2^ arm (median PFS: 11.2 months versus 7.6 months; hazard ratio [HR] = 0.69; 95% confidence interval (CI), 0.54–0.88; *P* = 0.0029; ORR: 62.9% versus 40.8%). A greater proportion of once-weekly Kd70 mg/m^2^ patients achieved a very good partial response or better (≥VGPR) and complete response or better (≥CR) compared with twice-weekly Kd27 mg/m^2^ patients (≥VGPR, 34.2% versus 13.4%; ≥CR, 7.0% versus 1.7%). Safety was generally comparable between the treatment arms^27^. Results from A.R.R.O.W. led to the recent approval of once-weekly Kd70 mg/m^2^ for the treatment of patients with RRMM^15,27^. Furthermore, compared with twice-weekly Kd27 mg/m^2^, once-weekly Kd70 mg/m^2^ improved treatment adherence, patient satisfaction, and health-related quality of life in patients with RRMM^13^. Overall, these results support the value of once-weekly carfilzomib as an additional, more convenient treatment option for patients with RRMM. Here we report results from a pre-planned subgroup analysis of A.R.R.O.W. to evaluate treatment effects by age, renal function, number of prior therapies, bortezomib-refractory status, ISS, and ECOG PS.

## Methods

### Study design and participants

Full A.R.R.O.W. study design details have been published previously^27^. Eligibility criteria included: two or three previous lines of therapy, prior exposure to a PI and an immunomodulatory drug (IMiD), refractory to most recent therapy, measurable disease (per International Myeloma Working Group [IMWG] consensus criteria^28,29^), ECOG PS of 0 or 1, and calculated or measured creatinine clearance (CrCL) of ≥30 mL/min. The primary endpoint of A.R.R.O.W. was PFS. Secondary endpoints included ORR, OS, and safety. The study protocol was approved by the institutional review boards or ethics committees of all participating sites, and all patients provided written informed consent.

### Procedures

Patients were randomized (1:1) to receive once-weekly Kd70 mg/m^2^ or twice-weekly Kd27 mg/m^2^. Treatment was given in 28-day cycles until disease progression, unacceptable toxicity, or withdrawal of consent. Patients in the once-weekly Kd70-mg/m^2^ group received carfilzomib on days 1, 8, and 15 (20 mg/m^2^ on day 1 [cycle 1]; 70 mg/m^2^ thereafter; 30-min intravenous infusion). Twice-weekly Kd27 mg/m^2^ patients received carfilzomib on days 1, 2, 8, 9, 15, and 16 (20 mg/m^2^ on days 1 and 2 during cycle 1; 27 mg/m^2^ thereafter; 10-min intravenous infusions). All patients received dexamethasone (40 mg) on days 1, 8, 15 (all cycles), and 22 (cycles 1–9 only).

### Subgroups

Patients in the intention-to-treat (ITT) population were grouped according to age (<65, 65–74, or ≥75 years), renal function (baseline CrCL <50, ≥50 to <80, or ≥80 mL/min), prior lines of therapy (2 or 3), bortezomib-refractory status (yes or no), ECOG PS (0 or 1), and ISS stage (stages 1 and 2 or stage 3). Within 21 days prior to randomization, adequate bone marrow and organ function assessments were performed at a central laboratory. Renal function was calculated using the Cockcroft and Gault formula as follows: [(140 − Age) × Mass (kg)/(72 × Creatinine (mg/dL)]; results were multiplied by 0.85 for female patients. Patients were considered refractory to bortezomib if (A) they were non-responsive to any regimen containing bortezomib (i.e., best overall response was stable or progressive disease) or (B) disease progression occurred within 60 days of bortezomib treatment discontinuation^27^. Here we report analyses of PFS, ORR, and safety in these prespecified subgroups. OS was not included because this data was not mature at the time of the interim analysis.

### Assessments

PFS, ORR, and best overall response were assessed in the ITT population. Response and disease progression were evaluated from the time of randomization in accordance with the IMWG Uniform Response Criteria^28,29^. Safety was assessed in all patients who received at least one dose of carfilzomib or dexamethasone.

### Statistical analyses

Median PFS was estimated using the Kaplan–Meier method. HRs and corresponding 95% CIs were estimated using an unstratified Cox proportional hazards model. Comparisons between treatment arms were evaluated using an unstratified log-rank test. The Clopper–Pearson method was used to estimate 95% CIs for ORR. Mantel–Haenszel unadjusted estimates were used to estimate the odds ratio (OR) and corresponding 95% CI. Comparisons between treatment arms were evaluated using Fisher exact test. Reported *P* values are one sided and unadjusted for multiple comparisons.

### Data sharing

Qualified researchers may request data from Amgen clinical studies. Complete details are available at the following: http://www.amgen.com/datasharing.

## Results

### Patient enrollment

The cutoff date for the pre-planned interim analysis was June 15, 2017^27^. Within the ITT population (*N* = 478), 240 patients received once-weekly Kd70 mg/m^2^ and 238 received twice-weekly Kd27 mg/m^2^. Baseline characteristics were generally balanced between treatment arms across subgroups. Select characteristics with ≥10% difference between treatment arms are shown in Supplementary Tables 1–4.

### Efficacy

#### Age

**Fig. 1:**
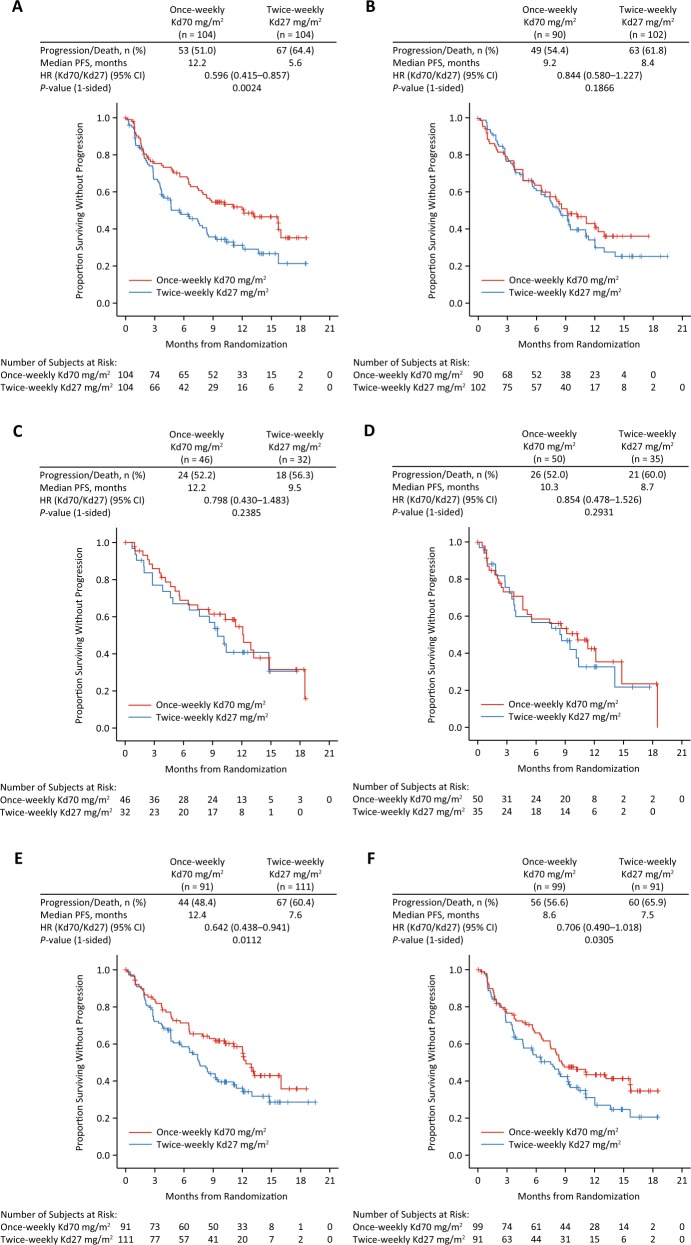
Kaplan–Meier curves for progression-free survival by subgroups’ age and renal function. **a** <65 years, **b** 65–74 years, **c** ≥75 years, **d** creatinine clearance (CrCL) <50 mL/min, **e** CrCL ≥50–<80 mL/min, **f** CrCL ≥80 mL/min.

In total, 208 patients (43.5%) were aged <65 years (once-weekly Kd70 mg/m^2^, *n* = 104; twice-weekly Kd27 mg/m^2^, *n* = 104), 192 patients (40.2%) were aged 65–74 years (*n* = 90; *n* = 102), and 78 (16.3%) patients were aged ≥75 years (*n* = 46; *n* = 32). The median PFS in patients aged <65 years was longer in the once-weekly Kd70-mg/m^2^ arm compared with the twice-weekly Kd27-mg/m^2^ group (12.2 versus 5.6 months; HR = 0.60; 95% CI, 0.42–0.86; *P* = 0.0024; Fig. 1). Among patients aged 65–74 and ≥75 years, median PFS (once-weekly Kd70 mg/m^2^ versus twice-weekly Kd27 mg/m^2^) was 9.2 versus 8.4 months (HR = 0.84; 95% CI, 0.58–1.23; *P* = 0.1866) and 12.2 versus 9.5 months (HR = 0.80; 95% CI, 0.43–1.48; *P* = 0.2385), respectively.

Response by age is shown in Table 1. ORR was improved with once-weekly Kd70 mg/m^2^ versus twice-weekly Kd27 mg/m^2^ for all age subgroups. ORRs (once-weekly Kd70 mg/m^2^ versus twice-weekly Kd27 mg/m^2^) were 64.4% versus 34.6% (OR, 3.42; 95% CI, 1.94–6.05) in patients aged <65 years, 60.0% versus 42.2% (OR, 2.06; 95% CI, 1.16–3.66) in patients aged 65–74 years, and 65.2% versus 56.3% (OR, 1.46; 95% CI, 0.58–3.68) in patients aged ≥75 years. Regardless of age, a higher proportion of patients treated with once-weekly Kd70 mg/m^2^ achieved a ≥VGPR and a ≥CR versus twice-weekly Kd27 mg/m^2^ (Table 1).

**Table 1:** Response rates by age and renal function subgroups.

Subgroup	Age						Baseline creatinine clearance					
	<65 years		65–74 years		≥75 years		<50 mL/min		≥50–<80 mL/min		≥80 mL/min	
	Once-weekly Kd70 mg/m^2^ (*n* = 104)	Twice-weekly Kd27 mg/m^2^ (*n* = 104)	Once-weekly Kd70 mg/m^2^ (*n* = 90)	Twice-weekly Kd27 mg/m^2^ (*n* = 102)	Once-weekly Kd70 mg/m^2^ (*n* = 46)	Twice-weekly Kd27 mg/m^2^ (*n* = 32)	Once-weekly Kd70 mg/m^2^ (*n* = 50)	Twice-weekly Kd27 mg/m^2^ (*n* = 35)	Once-weekly Kd70 mg/m^2^ (*n* = 91)	Twice-weekly Kd27 mg/m^2^ (*n* = 111)	Once-weekly Kd70 mg/m^2^ (*n* = 99)	Twice-weekly Kd27 mg/m^2^ (*n* = 91)
Best overall response, *n* (%)												
sCR	2 (1.9)	0	2 (2.2)	0	0	0	1 (2.0)	0	2 (2.2)	0	1 (1.0)	0
CR	7 (6.7)	4 (3.8)	5 (5.6)	0	1 (2.2)	0	1 (2.0)	0	5 (5.5)	1 (0.9)	7 (7.1)	3 (3.3)
VGPR	31 (29.8)	8 (7.7)	19 (21.1)	13 (12.7)	15 (32.6)	7 (21.9)	11 (22.0)	7 (20.0)	24 (26.4)	11 (9.9)	30 (30.3)	10 (11.0)
PR	27 (26.0)	24 (23.1)	28 (31.1)	30 (29.4)	14 (30.4)	11 (34.4)	12 (24.0)	7 (20.0)	29 (31.9)	32 (28.8)	28 (28.3)	26 (28.6)
ORR, *n* (%)	67 (64.4)	36 (34.6)	54 (60.0)	43 (42.2)	30 (65.2)	18 (56.3)	25 (50.0)	14 (40.0)	60 (65.9)	44 (39.6)	66 (66.7)	39 (42.9)
OR (95% CI)	3.420 (1.935–6.045)		2.058 (1.156–3.663)		1.458 (0.578–3.678)		1.500 (0.626–3.596)		2.947 (1.656–5.246)		2.667 (1.480–4.806)	
*P* value	<0.0001		0.0073		0.2412		0.1929		0.0001		0.0006	

*CI* confidence interval, *CR* complete response, *Kd27* carfilzomib (27 mg/m^2^) with dexamethasone, *Kd70* carfilzomib (70 mg/m^2^) with dexamethasone, *OR* odds ratio, *ORR* overall response rate, *PR* partial response, *sCR* stringent complete response, *VGPR* very good partial response.

#### Renal function

Eighty-five (17.8%) patients had baseline CrCL <50 mL/min (once-weekly Kd70 mg/m^2^, *n* = 50; twice-weekly Kd27 mg/m^2^, *n* = 35), 202 (42.3%) patients had baseline CrCL ≥50 to <80 mL/min (*n* = 91; *n* = 111), and 190 (39.7%) patients had baseline CrCL ≥80 mL/min (*n* = 99; *n* = 91). Median PFS (once-weekly Kd70 mg/m^2^ versus twice-weekly Kd27 mg/m^2^) was 10.3 versus 8.7 months (HR = 0.85; 95% CI, 0.48–1.53; *P* = 0.2931) in patients with CrCL <50 mL/min, 12.4 versus 7.6 months (HR = 0.64; 95% CI, 0.44–0.94; *P* = 0.0112) in patients with CrCL ≥50 to <80 mL/min, and 8.6 versus 7.5 months (HR = 0.71; 95% CI, 0.49–1.02; *P* = 0.0305) in patients with CrCL ≥80 mL/min (Fig. 1).

ORR was improved with once-weekly Kd70 mg/m^2^ across all renal function subgroups (Table 1). The ORRs for once-weekly Kd70 mg/m^2^ versus twice-weekly Kd27 mg/m^2^ were 50.0% versus 40.0% (OR, 1.50; 95% CI, 0.63–3.60) in patients with CrCL <50 mL/min, 65.9% versus 39.6% (OR, 2.95; 95% CI, 1.66–5.25) in patients with CrCL ≥50 to <80 mL/min, and 66.7% versus 42.9% (OR, 2.67; 95% CI, 1.48–4.81) in patients with CrCL ≥80 mL/min. A greater proportion of once-weekly Kd70-mg/m^2^ patients achieved ≥VGPR and ≥CR versus twice-weekly Kd27 mg/m^2^ across all renal function subgroups (Table 1).

#### Prior lines of therapy

**Fig. 2:**
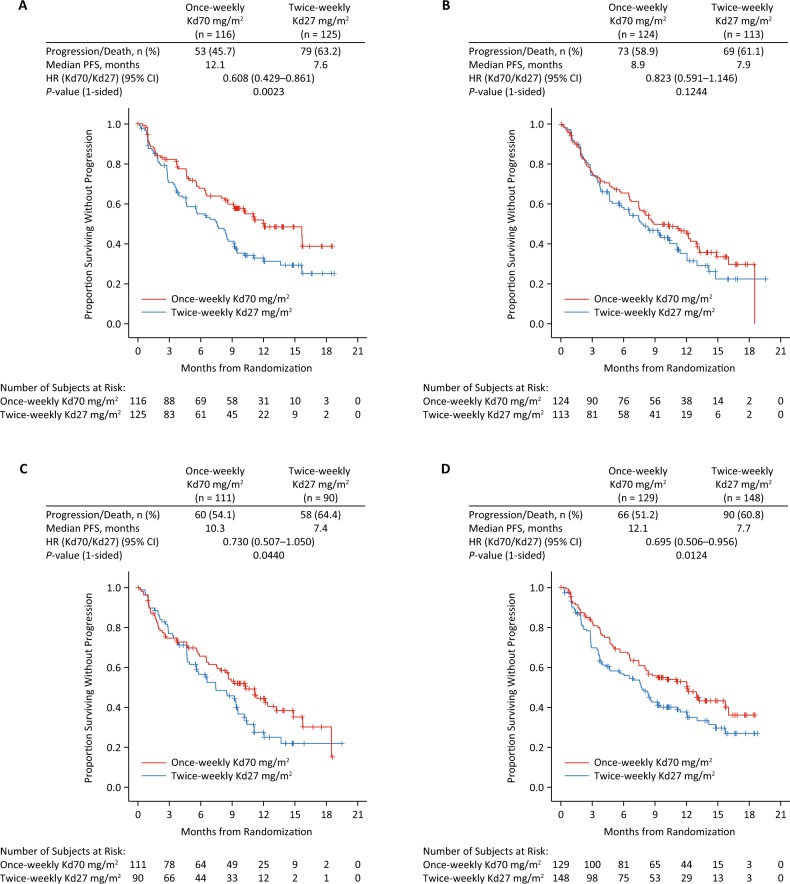
Kaplan–Meier curves for progression-free survival by prior lines of therapy and bortezomib-refractory status. **a** Two prior lines of therapy, **b** 3 prior lines of therapy, **c** refractory to bortezomib, **d** not refractory to bortezomib.

Within the ITT population, 241 (50.4%) patients received two prior therapies (once-weekly Kd70 mg/m^2^, *n* = 116; twice-weekly Kd27 mg/m^2^, *n* = 125) and 237 (49.6%) patients received three prior therapies (*n* = 124; *n* = 113). Median PFS was comparable or better in patients treated with once-weekly Kd70 mg/m^2^ vs twice-weekly Kd27 mg/m^2^ according to prior number of therapies. Median PFS (once-weekly Kd70 mg/m^2^ versus twice-weekly Kd27 mg/m^2^) was 12.1 versus 7.6 months (HR = 0.61; 95% CI, 0.43–0.86; *P* = 0.0023) in the two prior therapies subgroup and 8.9 versus 7.9 months (HR = 0.82; 95% CI, 0.59–1.15; *P* = 0.1244) in the three prior therapy subgroup (Fig. 2).

ORRs for once-weekly Kd70 mg/m^2^ versus twice-weekly Kd27 mg/m^2^ were 62.9% versus 40.8% (OR, 2.46; 95% CI, 1.47–4.14) in the two prior line subgroup and 62.9% versus 40.7% (OR, 2.47; 95% CI, 1.46–4.17) in the three prior line subgroup, respectively. Patients treated with once-weekly Kd70 mg/m^2^ achieved higher rates of ≥VGPR and ≥CR versus those treated with twice-weekly Kd27 mg/m^2^ regardless of the number of prior lines of therapy (Table 2).

**Table 2:** Response rates by prior lines of therapy and bortezomib-refractory status subgroups.

Subgroup	Prior lines of therapy				Bortezomib-refractory status			
	2 prior lines		3 prior lines		Refractory to bortezomib		Not refractory to bortezomib	
	Once-weekly Kd70 mg/m^2^ (*n* = 116)	Twice-weekly Kd27 mg/m^2^ (*n* = 125)	Once-weekly Kd70 mg/m^2^ (*n* = 124)	Twice-weekly Kd27 mg/m^2^ (*n* = 113)	Once-weekly Kd70 mg/m^2^ (*n* = 111)	Twice-weekly Kd27 mg/m^2^ (*n* = 90)	Once-weekly Kd70 mg/m^2^ (*n* = 129)	Twice-weekly Kd27 mg/m^2^ (*n* = 148)
Best overall response, *n* (%)								
sCR	1 (0.9)	0	3 (2.4)	0	0	0	4 (3.1)	0
CR	10 (8.6)	4 (3.2)	3 (2.4)	0	7 (6.3)	2 (2.2)	6 (4.7)	2 (1.4)
VGPR	32 (27.6)	14 (11.2)	33 (26.6)	14 (12.4)	23 (20.7)	4 (4.4)	42 (32.6)	24 (16.2)
PR	30 (25.9)	33 (26.4)	39 (31.5)	32 (28.3)	34 (30.6)	32 (35.6)	35 (27.1)	33 (22.3)
ORR, *n* (%)	73 (62.9)	51 (40.8)	78 (62.9)	46 (40.7)	64 (57.7)	38 (42.2)	87 (67.4)	59 (39.9)
OR (95% CI)	2.463 (1.466–4.139)		2.470 (1.464–4.167)		1.863 (1.061–3.271)		3.125 (1.907–5.120)	
*P* value	0.0004		0.0004		0.0169		<0.0001	

*CI* confidence interval, *CR* complete response, *Kd27* carfilzomib (27 mg/m^2^) with dexamethasone, *Kd70* carfilzomib (70 mg/m^2^) with dexamethasone, *OR* odds ratio, *ORR* overall response rate, *PR* partial response, *sCR* stringent complete response, *VGPR* very good partial response.

#### Bortezomib-refractory status

Within the ITT population, 201 (42.1%) patients were bortezomib refractory (once-weekly Kd70 mg/m^2^, *n* = 111; twice-weekly Kd27 mg/m^2^, *n* = 90) and 277 (57.9%) patients were not bortezomib refractory (*n* = 129; *n* = 148). Median PFS (once-weekly Kd70 mg/m^2^ versus twice-weekly Kd27 mg/m^2^) was 10.3 versus 7.4 months (HR = 0.73; 95% CI, 0.51–1.05; *P* = 0.0440) in patients with disease refractory to bortezomib and 12.1 versus 7.7 months (HR = 0.70; 95% CI, 0.51–0.96; *P* = 0.0124) in patients with disease not refractory to bortezomib (Fig. 2). Twice-weekly Kd27 mg/m^2^ had similar median PFS outcomes, irrespective of disease sensitivity to bortezomib.

Patients treated with once-weekly Kd70 mg/m^2^ demonstrated higher response rates compared with those treated with twice-weekly Kd27 mg/m^2^ across subgroups based on disease sensitivity to bortezomib. ORRs (once-weekly Kd70 mg/m^2^ versus twice-weekly Kd27 mg/m^2^) were 57.7% versus 42.2% (OR, 1.86; 95% CI, 1.06–3.27) in patients with bortezomib-refractory disease and 67.4% versus 39.9% (OR, 3.13; 95% CI, 1.91–5.12) in patients with disease not refractory to bortezomib. Regardless of bortezomib-refractory status, a greater proportion of once-weekly Kd70-mg/m^2^ patients achieved ≥VGPR and ≥CR versus twice-weekly Kd27-mg/m^2^ patients (Table 2).

### Safety

#### Age

The median number of cycles patients received carfilzomib (once-weekly Kd70 mg/m^2^ and twice-weekly Kd27 mg/m^2^) was 11 and 6 cycles in patients aged <65 years, 9 and 9 cycles in patients aged 65–74 years, and 10 and 10 cycles in patients aged ≥75 years, respectively. Median carfilzomib treatment duration was higher in the once-weekly Kd70-mg/m^2^ treatment arm relative to the twice-weekly Kd27-mg/m^2^ treatment arm in patients aged <65 years (41.1 and 21.3 weeks, respectively); median treatment duration was 33.1 and 34.9 weeks in patients aged 65–74 years, and 40.1 and 37.3 weeks in patients aged ≥75 years, respectively. Treatment-emergent adverse events (TEAEs) by age are shown in Table 3. The incidence of grade ≥3 TEAEs (once-weekly Kd70 mg/m^2^ and twice-weekly Kd27 mg/m^2^) was 59.2% and 56.3% (<65 years), 68.9% and 63.4% (65–74 years), and 84.4% and 74.2% (≥75 years), respectively. Rates of grade ≥3 cardiac failure (once-weekly Kd70 mg/m^2^ and twice-weekly Kd27 mg/m^2^) were 1.0% and 5.8%, 5.6% and 2.0%, and 2.2% and 6.5% in these age subgroups, respectively.

**Table 3:** Safety in once-weekly Kd70-mg/m^2^ versus twice-weekly Kd27-mg/m^2^ treatment arms by age and renal function subgroups.

Subgroup	Age						Baseline creatinine clearance					
	<65 years		65–74 years		≥75 years		<50 mL/min		≥50–<80 mL/min		≥80 mL/min	
	Once-weekly Kd70 mg/m^2^ (*n* = 103)	Twice-weekly Kd27 mg/m^2^ (*n* = 103)	Once-weekly Kd70 mg/m^2^ (*n* = 90)	Twice-weekly Kd27 mg/m^2^ (*n* = 101)	Once-weekly Kd70 mg/m^2^ (*n* = 45)	Twice-weekly Kd27 mg/m^2^ (*n* = 31)	Once-weekly Kd70 mg/m^2^ (*n* = 49)	Twice-weekly Kd27 mg/m^2^ (*n* = 34)	Once-weekly Kd70 mg/m^2^ (*n* = 90)	Twice-weekly Kd27 mg/m^2^ (*n* = 109)	Once-weekly Kd70 mg/m^2^ (*n* = 99)	Twice-weekly Kd27 mg/m^2^ (*n* = 91)
Any grade TEAEs, *n* (%)	95 (92.2)	100 (97.1)	87 (96.7)	98 (97.0)	45 (100.0)	31 (100.0)	47 (95.9)	33 (97.1)	89 (98.9)	106 (97.2)	91 (91.9)	89 (97.8)
Grade ≥3 TEAEs, *n* (%)	61 (59.2)	58 (56.3)	62 (68.9)	64 (63.4)	38 (84.4)	23 (74.2)	38 (77.6)	24 (70.6)	64 (71.1)	67 (61.5)	59 (59.6)	54 (59.3)
Grade ≥3 TEAEs of interest, *n* (%)												
Peripheral neuropathy	0	1 (1.0)	0	0	0	0	0	0	0	1 (0.9)	0	0
Acute renal failure	2 (1.9)	5 (4.9)	7 (7.8)	8 (7.9)	0	0	5 (10.2)	2 (5.9)	3 (3.3)	7 (6.4)	1 (1.0)	4 (4.4)
Acute kidney injury	1 (1.0)	3 (2.9)	7 (7.8)	5 (5.0)	0	0	4 (8.2)	0	3 (3.3)	5 (4.6)	1 (1.0)	3 (3.3)
Cardiac failure	1 (1.0)	6 (5.8)	5 (5.6)	2 (2.0)	1 (2.2)	2 (6.5)	1 (2.0)	2 (5.9)	6 (6.7)	4 (3.7)	0	4 (4.4)
Ischemic heart disease	1 (1.0)	0	0	1 (1.0)	1 (2.2)	1 (3.2)	1 (2.0)	1 (2.9)	0	0	1 (1.0)	1 (1.1)
Pulmonary hypertension	0	1 (1.0)	0	0	0	0	0	1 (2.9)	0	0	0	0
Hypertension	4 (3.9)	2 (1.9)	4 (4.4)	7 (6.9)	6 (13.3)	4 (12.9)	5 (10.2)	2 (5.9)	5 (5.6)	8 (7.3)	4 (4.0)	3 (3.3)
Anemia	17 (16.5)	23 (22.3)	15 (16.7)	18 (17.8)	10 (22.2)	1 (3.2)	16 (32.7)	9 (26.5)	14 (15.6)	19 (17.4)	12 (12.1)	14 (15.4)
Thrombocytopenia	8 (7.8)	9 (8.7)	6 (6.7)	6 (5.9)	3 (6.7)	1 (3.2)	5 (10.2)	3 (8.8)	5 (5.6)	6 (5.5)	7 (7.1)	7 (7.7)
Neutropenia	7 (6.8)	10 (9.7)	3 (3.3)	4 (4.0)	4 (8.9)	2 (6.5)	4 (8.2)	2 (5.9)	6 (6.7)	5 (4.6)	4 (4.0)	9 (9.9)
TEAEs leading to carfilzomib discontinuation, *n* (%)	7 (6.8)	14 (13.6)	15 (16.7)	8 (7.9)	8 (17.8)	5 (16.1)	14 (28.6)	5 (14.7)	13 (14.4)	12 (11.0)	3 (3.0)	10 (11.0)
TEAEs leading to dexamethasone discontinuation, *n* (%)	9 (8.7)	14 (13.6)	17 (18.9)	8 (7.9)	9 (20.0)	5 (16.1)	15 (30.6)	5 (14.7)	15 (16.7)	12 (11.0)	5 (5.1)	10 (11.0)
TEAEs leading to death, *n* (%)	10 (9.7)	9 (8.7)	10 (11.1)	5 (5.0)	2 (4.4)	4 (12.9)	9 (18.4)	6 (17.6)	6 (6.7)	5 (4.6)	7 (7.1)	7 (7.7)

TEAEs are defined as any adverse event with an onset date from the first dose through 30 days after the last dose of any study drug.

Adverse events were coded using MedDRA version 20.0 and graded using NCI-CTCAE (version 4.03).

Subjects were counted only once for each search strategy and each preferred term.

Adverse events (peripheral neuropathy, cardiac failure, ischemic heart disease, and pulmonary hypertension) are listed as SMQ, narrow scope or preferred terms (acute kidney injury, anemia, thrombocytopenia, and neutropenia).

*Kd27* carfilzomib (27 mg/m^2^) with dexamethasone, *Kd70* carfilzomib (70 mg/m^2^) with dexamethasone, *MedDRA* Medical Dictionary for Regulatory Activities, *NCI-CTCAE* National Cancer Institute-Common Terminology Criteria for Adverse Events, *SMQ* Standardized MedDRA Queries, *TEAE* treatment-emergent adverse event.

TEAEs leading to carfilzomib discontinuation (once-weekly Kd70 mg/m^2^ and twice-weekly Kd27 mg/m^2^) occurred at rates of 6.8% and 13.6% (<65 years), 16.7% and 7.9% (65–74 years), and 17.8% and 16.1% (≥75 years); rates of TEAEs leading to dexamethasone discontinuation were 8.7% and 13.6%, 18.9% and 7.9%, and 20.0% and 16.1%, respectively. Incidence of fatal TEAEs (once-weekly Kd70 mg/m^2^ and twice-weekly Kd27 mg/m^2^) was 9.7% and 8.7% (<65 years), 11.1% and 5.0% (65–74 years), and 4.4% and 12.9% (≥75 years).

#### Renal function

TEAEs by renal function are shown in Table 3. Rates of grade ≥3 TEAEs by baseline CrCL (once-weekly Kd70 mg/m^2^ and twice-weekly Kd27 mg/m^2^) were 77.6% and 70.6% (CrCL < 50 mL/min), 71.1% and 61.5% (CrCL ≥ 50 to <80 mL/min), and 59.6% and 59.3% (CrCL ≥ 80 mL/min). The incidence of grade ≥3 cardiac failure (once-weekly Kd70 mg/m^2^ and twice-weekly Kd27 mg/m^2^) was 2.0% and 5.9%, 6.7% and 3.7%, and 0% and 4.4% in these renal function subgroups, respectively.

TEAEs leading to carfilzomib discontinuation (once-weekly Kd70 mg/m^2^ and twice-weekly Kd27 mg/m^2^) occurred at rates of 28.6% and 14.7% (CrCL < 50 mL/min), 14.4% and 11.0% (CrCL ≥ 50 to <80 mL/min), and 3.0% and 11.0% (CrCL ≥ 80 mL/min); rates of TEAEs leading to dexamethasone discontinuation were 30.6% and 14.7%, 16.7% and 11.0%, and 5.1% and 11.0%, respectively. Incidence of fatal TEAEs (once-weekly Kd70 mg/m^2^ and twice-weekly Kd27 mg/m^2^) was 18.4% and 17.6% (CrCL < 50 mL/min), 6.7% and 4.6% (CrCL ≥ 50 to <80 mL/min), and 7.1% and 7.7% (CrCL ≥ 80 mL/min).

#### Prior lines of therapy

TEAEs by prior lines of therapy are shown in Table 4. The incidence of grade ≥3 TEAEs (once-weekly Kd70 mg/m^2^ and twice-weekly Kd27 mg/m^2^) was 59.1% and 65.0% (2 prior lines) and 75.6% and 58.0% (3 prior lines). The incidence of grade ≥3 cardiac failure (once-weekly Kd70 mg/m^2^ and twice-weekly Kd27 mg/m^2^) was 2.6% and 2.4% (2 prior lines) and 3.3% and 6.3% (3 prior lines).

**Table 4:** Safety in once-weekly Kd70-mg/m^2^ versus twice-weekly Kd27-mg/m^2^ treatment arms by prior lines of therapy and bortezomib-refractory status subgroups.

Subgroup	Prior lines of therapy				Bortezomib-refractory status			
	2 prior lines		3 prior lines		Refractory to bortezomib		Not refractory to bortezomib	
	Once-weekly Kd70 mg/m^2^ (*n* = 115)	Twice-weekly Kd27 mg/m^2^ (*n* = 123)	Once-weekly Kd70 mg/m^2^ (*n* = 123)	Twice-weekly Kd27 mg/m^2^ (*n* = 112)	Once-weekly Kd70 mg/m^2^ (*n* = 110)	Twice-weekly Kd27 mg/m^2^ (*n* = 89)	Once-weekly Kd70 mg/m^2^ (*n* = 128)	Twice-weekly Kd27 mg/m^2^ (*n* = 146)
Any grade TEAEs, *n* (%)	107 (93.0)	121 (98.4)	120 (97.6)	108 (96.4)	104 (94.5)	88 (98.9)	123 (96.1)	141 (96.6)
Grade ≥3 TEAEs, *n* (%)	68 (59.1)	80 (65.0)	93 (75.6)	65 (58.0)	75 (68.2)	55 (61.8)	86 (67.2)	90 (61.6)
Grade ≥3 TEAEs of interest, *n* (%)								
Peripheral neuropathy	0	0	0	1 (0.9)	0	0	0	1 (0.7)
Acute renal failure	3 (2.6)	6 (4.9)	6 (4.9)	7 (6.3)	4 (3.6)	3 (3.4)	5 (3.9)	10 (6.8)
Acute kidney injury	3 (2.6)	6 (4.9)	5 (4.1)	2 (1.8)	3 (2.7)	2 (2.2)	5 (3.9)	6 (4.1)
Cardiac failure	3 (2.6)	3 (2.4)	4 (3.3)	7 (6.3)	5 (4.5)	6 (6.7)	2 (1.6)	4 (2.7)
Ischemic heart disease	2 (1.7)	0	0	2 (1.8)	1 (0.9)	1 (1.1)	1 (0.8)	1 (0.7)
Pulmonary hypertension	0	0	0	1 (0.9)	0	1 (1.1)	0	0
Hypertension	3 (2.6)	8 (6.5)	11 (8.9)	5 (4.5)	6 (5.5)	7 (7.9)	8 (6.3)	6 (4.1)
Anemia	16 (13.9)	19 (15.4)	26 (21.1)	23 (20.5)	24 (21.8)	16 (18.0)	18 (14.1)	26 (17.8)
Thrombocytopenia	7 (6.1)	9 (7.3)	10 (8.1)	7 (6.3)	10 (9.1)	7 (7.9)	7 (5.5)	9 (6.2)
Neutropenia	5 (4.3)	10 (8.1)	9 (7.3)	6 (5.4)	7 (6.4)	7 (7.9)	7 (5.5)	9 (6.2)
TEAEs leading to carfilzomib discontinuation, *n* (%)	15 (13.0)	12 (9.8)	15 (12.2)	15 (13.4)	16 (14.5)	9 (10.1)	14 (10.9)	18 (12.3)
TEAEs leading to dexamethasone discontinuation, *n* (%)	19 (16.5)	12 (9.8)	16 (13.0)	15 (13.4)	16 (14.5)	9 (10.1)	19 (14.8)	18 (12.3)
TEAEs leading to death, *n* (%)	10 (8.7)	8 (6.5)	12 (9.8)	10 (8.9)	7 (6.4)	7 (7.9)	15 (11.7)	11 (7.5)

TEAEs are defined as any adverse event with an onset date from the first dose through 30 days after the last dose of any study drug.

Adverse events were coded using MedDRA version 20.0 and graded using NCI-CTCAE (version 4.03).

Subjects were counted only once for each search strategy and each preferred term.

Adverse events (peripheral neuropathy, cardiac failure, ischemic heart disease, and pulmonary hypertension) are listed as SMQ, narrow scope or preferred terms (acute kidney injury, anemia, thrombocytopenia, and neutropenia).

*Kd27* carfilzomib (27 mg/m^2^) with dexamethasone, *Kd70* carfilzomib (70 mg/m^2^) with dexamethasone, *MedDRA* Medical Dictionary for Regulatory Activities, *NCI-CTCAE* National Cancer Institute-Common Terminology Criteria for Adverse Events, *SMQ* Standardized MedDRA Queries, *TEAE* treatment-emergent adverse event.

TEAEs leading to carfilzomib discontinuation (once-weekly Kd70 mg/m^2^ and twice-weekly Kd27 mg/m^2^) occurred at rates of 13.0% and 9.8% (2 prior lines) and 12.2% and 13.4% (3 prior lines); rates of TEAEs leading to dexamethasone discontinuation were 16.5% and 9.8% (2 prior lines) and 13.0% and 13.4% (3 prior lines). The incidence of fatal TEAEs (once-weekly Kd70 mg/m^2^ and twice-weekly Kd27 mg/m^2^) was 8.7% and 6.5% (2 prior lines) and 9.8% and 8.9% (3 prior lines).

#### Bortezomib-refractory status

Rates of TEAEs by bortezomib-refractory status are shown in Table 4. Frequencies of grade ≥3 TEAEs (once-weekly Kd70 mg/m^2^ and twice-weekly Kd27 mg/m^2^) were 68.2% and 61.8% (refractory to bortezomib) and 67.2% and 61.6% (not refractory to bortezomib). The frequencies of grade ≥3 cardiac failure (once-weekly Kd70 mg/m^2^ and twice-weekly Kd27 mg/m^2^) by bortezomib-refractory status were 4.5% and 6.7% (refractory to bortezomib) and 1.6% and 2.7% (not refractory to bortezomib).

TEAEs leading to carfilzomib discontinuation (once-weekly Kd70 mg/m^2^ and twice-weekly Kd27 mg/m^2^) occurred at rates of 14.5% and 10.1% (refractory to bortezomib) and 10.9% and 12.3% (not refractory to bortezomib); frequencies of TEAEs leading to dexamethasone discontinuation were 14.5% and 10.1% (refractory to bortezomib) and 14.8% and 12.3% (not refractory to bortezomib). Rates of fatal TEAEs (once-weekly Kd70 mg/m^2^ and twice-weekly Kd27 mg/m^2^) were 6.4% and 7.9% (refractory to bortezomib) and 11.7% and 7.5% (not refractory to bortezomib).

#### ECOG PS and ISS stage

Rates of grade ≥3 TEAEs of interest are shown in Supplementary Table 5. The incidence of grade ≥3 cardiac failure (once-weekly Kd70 mg/m^2^ and twice-weekly Kd27 mg/m^2^) by ECOG PS was 1.7% and 2.6% (ECOG PS of 0) and 4.2% and 5.9% (ECOG PS of 1). The rates of grade ≥3 cardiac failure (once-weekly Kd70 mg/m^2^ and twice-weekly Kd27 mg/m^2^) by ISS stage were 2.3% and 4.0% (ISS stages 1 and 2) and 4.8% and 3.7% (ISS stage 3).

## Discussion

In this pre-planned subgroup analysis of the A.R.R.O.W. study, patients were evaluated by age, renal function, prior lines of therapy, bortezomib-refractory status, ECOG PS, and ISS stage. These factors have demonstrated prognostic significance in RRMM^6–10^ and are important considerations when selecting therapy. Across nearly all examined subgroups in A.R.R.O.W., once-weekly administration of carfilzomib at the higher 70-mg/m^2^ dose in combination with dexamethasone was associated with longer median PFS and higher ORR compared with twice-weekly administration of carfilzomib at the 27-mg/m^2^ dose in combination with dexamethasone. These findings are consistent with those in the overall population^27^. Part of the observed benefit in patients receiving once-weekly Kd70 mg/m^2^ in A.R.R.O.W. is likely due to the higher dose of carfilzomib administered in this treatment arm. Furthermore, patient-reported outcomes analysis reinforced that the higher 70-mg/m^2^ carfilzomib dose is convenient and provided more favorable health-related quality of life than the 27-mg/m^2^ carfilzomib dose^13^. Adherence to therapy might have improved for patients treated with once-weekly Kd70 mg/m^2^ due to the more convenient weekly carfilzomib dosing schedule, which could have translated to improved clinical outcomes.

Elderly patients with MM are a challenging population to treat, in part due to higher chemotherapy toxicities^30^ and comorbidity burden^31,32^. In ENDEAVOR and ASPIRE, analyses of twice-weekly carfilzomib-based therapies by age demonstrated consistent improvements in PFS and OS compared with the control treatment arms across all subgroups^20,21^. In A.R.R.O.W., once-weekly Kd70 mg/m^2^ improved PFS and ORR for all age subgroups versus twice-weekly Kd27 mg/m^2^. Compared with the two older-age subgroups, patients in the younger subgroup (aged <65 years) received a greater median number of cycles of treatment in the once-weekly Kd70 mg/m^2^ arm than in the twice-weekly Kd27 mg/m^2^ arm. Since patients aged <65 years stayed on treatment longer with once-weekly Kd70 mg/m^2^ compared with twice-weekly Kd27 mg/m^2^ relative to the older-age subgroups, the younger-age patients receiving once-weekly Kd70 mg/m^2^ were able to derive a greater PFS benefit.

The safety profile for once-weekly Kd70 mg/m^2^ was generally comparable to twice-weekly Kd27 mg/m^2^ across all assessed age subgroups. In patients aged <65 years, the shorter median duration of carfilzomib administration in the twice-weekly Kd27 mg/m^2^ arm (21.3 weeks) compared to once-weekly Kd70 mg/m^2^ (41.1 weeks) may be partially explained by the higher incidence of disease progression or death in the twice-weekly Kd27-mg/m^2^ arm (64.4%) compared to the once-weekly Kd70-mg/m^2^ arm (51.0%) (Fig. 1a). In patients aged ≥75 years, the higher incidence of grade ≥3 TEAEs in the once-weekly Kd70-mg/m^2^ group (84.4%) compared to those in the twice-weekly Kd27-mg/m^2^ group (74.2%) may be due to the higher proportion of patients with baseline CrCL 30–<50 mL/min (56.5% versus 40.6%). Overall, once-weekly Kd70 mg/m^2^ was effective and well tolerated in patients with RRMM regardless of age. The improved convenience of the once-weekly dosing schedule could be important for elderly patients with restricted mobility or for those who are working.

Impaired renal function is a common clinicopathological feature of MM that has been associated with worse prognosis and survival in patients^8^. Furthermore, drug dosing can be complicated by renal impairment, which can increase or worsen AEs^33^. In a subgroup analysis of ENDEAVOR, twice-weekly Kd56 therapy demonstrated clinically meaningful improvements in PFS and OS across renal subgroups, including severe renal impairment, compared with bortezomib-based therapy^26^. Our results show that patients administered once-weekly Kd70 mg/m^2^ had longer median PFS and higher response rates compared with twice-weekly Kd27 mg/m^2^ across all renal function subgroups, including patients with baseline CrCL <50 mL/min.

In patients with baseline CrCL <80 mL/min, rates of grade ≥3 TEAEs and TEAEs leading to treatment discontinuation were greater in the once-weekly Kd70-mg/m^2^ group compared to the twice-weekly Kd27-mg/m^2^ group. Specifically in patients with CrCL <50 mL/min, the incidence of TEAEs leading to carfilzomib treatment discontinuation was 28.6% for once-weekly Kd70 mg/m^2^ compared with 14.7% for twice-weekly Kd27 mg/m^2^. Similar rates of grade ≥3 heart failure, hypertension, and acute kidney injury were reported for once-weekly Kd70 mg/m^2^ and twice-weekly Kd27 mg/m^2^ within each renal subgroup. Overall, once-weekly Kd70 mg/m^2^ had a favorable benefit–risk profile relative to twice-weekly Kd27 mg/m^2^ in patients with baseline CrCL ≥50 mL/min. In patients with decreased renal function (CrCL < 50 mL/min), the once-weekly Kd70-mg/m^2^ regimen improved PFS and response rates compared with the twice-weekly Kd27-mg/m^2^ regimen; however, a higher rate of AEs leading to carfilzomib treatment discontinuation was observed with once-weekly Kd70 mg/m^2^. Taken together, once-weekly Kd70 mg/m^2^ demonstrates benefit over twice-weekly Kd27 mg/m^2^, although to a lesser extent in patients with CrCL <50 mL/min.

Previously treated patients with RRMM are a challenging population to treat, as the disease has been reported to become less sensitive or refractory to certain therapies with each successive line of treatment^6,34^. Previous subgroup reports from ASPIRE^22^ and ENDEAVOR^23^ demonstrated improved PFS and ORR of twice-weekly carfilzomib-based regimens compared to recent SOCs, regardless of the number of prior therapies patients had received before enrollment. Consistent with previous reports^22,23^, a greater benefit was observed in patients with fewer previous therapies, suggesting that carfilzomib efficacy (administered once- or twice-weekly) can be optimized by earlier administration in the disease course for patients with RRMM.

The safety profiles for the prior lines subgroups were generally consistent with those reported for the overall population. In patients previously treated with three lines of therapy, the incidence of grade ≥3 TEAEs was greater in the once-weekly Kd70-mg/m^2^ treatment arm (75.6%) compared with the twice-weekly Kd27-mg/m^2^ arm (58.0%). This may be partially due to the higher proportion of patients aged 75–84 years (22.6% versus 11.5%) in the once-weekly Kd70-mg/m^2^ treatment arm. Importantly, the incidence of grade ≥3 cardiac failure was <7% across treatment arms and was lower for once-weekly Kd70 mg/m^2^ (2.6%–3.3%), and no additional toxicities were found. This subgroup analysis confirmed that once-weekly treatment with carfilzomib (70 mg/m^2^) plus dexamethasone is safe, feasible, and superior to twice-weekly carfilzomib (27 mg/m^2^) plus dexamethasone, regardless of the number of prior therapies.

Bortezomib is a common component of frontline therapies, and there is a need for effective salvage options in patients whose disease becomes refractory to this agent. The ASPIRE trial previously reported improved PFS and OS in bortezomib-refractory patients treated with twice-weekly KRd versus Rd^18,22^. In A.R.R.O.W., patients treated with once-weekly Kd70 mg/m^2^ demonstrated longer median PFS and higher ORRs versus those treated with twice-weekly Kd27 mg/m^2^, regardless of disease sensitivity to bortezomib. In both treatment arms, absolute PFS durations and response rates were lower in patients with bortezomib-refractory disease compared with bortezomib-sensitive disease. This is consistent with the existence of some cross-resistance between the two PIs^35,36^. Taken together with ASPIRE, these results support the benefit of carfilzomib-based therapy for patients with disease refractory to bortezomib.

Safety profiles by bortezomib-refractory status were comparable to the overall population^27^. Once-weekly Kd70 mg/m^2^ demonstrated improved efficacy with a similar safety profile compared to twice-weekly Kd27 mg/m^2^, regardless of bortezomib-refractory status. As continuous treatment with lenalidomide has become a new SOC in frontline MM^37,38^, there is a need for active regimens to treat patients who have relapsed or become refractory to lenalidomide. In the A.R.R.O.W. trial, 401 (83.9%) patients had prior lenalidomide exposure and 356 (74.5%) were refractory to any prior lenalidomide^27^. Although lenalidomide-exposed and lenalidomide-refractory patient subgroups were not evaluated in this study, analyses from these subgroups are underway and will be presented in a separate paper.

ECOG PS (0–5) and ISS stages (1–3) are prognostic factors used to assess patients with MM^39,40^, with higher values associated with worse prognosis and greater susceptibility to AEs. In our study, grade ≥3 TEAEs, including cardiac TEAEs, were comparable across treatment arms, regardless of ECOG PS (0 or 1) or ISS stage (stage 1 and 2 or stage 3). Once-weekly Kd70 mg/m^2^, therefore, represents a tolerable therapeutic option in RRMM patients with advanced disease and functional impairment.

Limitations of this subgroup analysis of A.R.R.O.W. include the open-label trial design and the small numbers of patients in subgroups. In addition, the control group in our study was administered twice-weekly carfilzomib at a dose of 27 mg/m^2^ in combination with dexamethasone; the currently approved dose of carfilzomib in combination with dexamethasone is 56 mg/m^2^ (based on ENDEAVOR^15,16^), which was not yet approved during the enrollment period of the A.R.R.O.W. study. Nevertheless, findings from this study warrant further investigation.

Clinical practice guidelines from the National Comprehensive Cancer Network, American Society of Oncology, and Cancer Care Ontario recommend doublet and triplet regimens as treatment options for patients with previously treated MM^41,42^. In randomized trials, carfilzomib- and daratumumab-based triplets have improved PFS, ORR, and/or OS in relapsed and/or refractory MM patients relative to doublet therapies; however, triplet therapies were also associated with higher rates of toxicity in these studies^17,18,43–46^. Therefore, patients with lower tolerance for increased toxicity, higher comorbidity burden, and/or frail status may not be suited for triplet therapies^42^. Comparisons of once-weekly Kd70 mg/m^2^ with currently available data from triplet combination studies are challenging, given differences in patient population (e.g., prior therapy, sensitivity to IMiDs and/or PIs, baseline creatinine clearance) and stratification of subgroups^17,20,22,43–47^. Future studies evaluating the efficacy and safety of once-weekly Kd70 mg/m^2^ dosing relative to recommended triplet salvage regimens may further highlight the potential utility of once-weekly Kd70 mg/m^2^ in the RRMM treatment armamentarium.

## Conclusions

In this pre-planned subgroup analysis of the A.R.R.O.W. study, PFS and ORR were consistently improved in the once-weekly carfilzomib treatment arm at the higher 70-mg/m^2^ dose compared with the twice-weekly carfilzomib treatment arm at the 27-mg/m^2^ dose across several important baseline patient and disease characteristics. The safety profiles of once-weekly Kd70 mg/m^2^ in each patient subgroup were generally consistent with that in the overall population^27^. Overall, this subgroup analysis of A.R.R.O.W. supports the favorable benefit–risk profile of once-weekly Kd70 mg/m^2^ and the use of this regimen as a safe, effective, and convenient treatment option for patients with RRMM, regardless of age, prior lines of therapy, and bortezomib-refractory status.

## Supplementary information

Supplemental Material

## References

[1] Kumar SK (2017). Multiple myeloma. Nat. Rev. Dis. Primers.

[2] Ferlay J (2015). Cancer incidence and mortality worldwide: sources, methods and major patterns in GLOBOCAN 2012. Int. J. Cancer.

[3] Cook G, Zweegman S, Mateos MV, Suzan F, Moreau P (2018). A question of class: treatment options for patients with relapsed and/or refractory multiple myeloma. Crit. Rev. Oncol. Hematol..

[4] Sonneveld P (2016). Treatment of multiple myeloma with high-risk cytogenetics: a consensus of the International Myeloma Working Group. Blood.

[5] Dingli D (2017). Therapy for relapsed multiple myeloma: guidelines from the Mayo stratification for myeloma and risk-adapted therapy. Mayo Clin. Proc..

[6] Kumar SK (2004). Clinical course of patients with relapsed multiple myeloma. Mayo Clin. Proc..

[7] Tandon N (2017). Clinical utility of the Revised International Staging System in unselected patients with newly diagnosed and relapsed multiple myeloma. Blood Cancer J..

[8] Eleutherakis-Papaiakovou V (2007). Renal failure in multiple myeloma: incidence, correlations, and prognostic significance. Leuk. Lymphoma.

[9] Willan J (2016). Multiple myeloma in the very elderly patient: challenges and solutions. Clin. Interv. Aging.

[10] Laubach J (2016). Management of relapsed multiple myeloma: recommendations of the International Myeloma Working Group. Leukemia.

[11] Kim H (2011). Efficacy and safety of once-weekly bortezomib infusion in the treatment of relapsed/refractory multiple myeloma. Blood.

[12] Hainsworth JD (2008). Weekly treatment with bortezomib for patients with recurrent or refractory multiple myeloma: a phase 2 trial of the Minnie Pearl Cancer Research Network. Cancer.

[13] Moreau Philippe, Kumar Shaji, Boccia Ralph, Iida Shinsuke, Goldschmidt Hartmut, Cocks Kim, Trigg Andrew, Zahlten-Kumeli Anita, Yucel Emre, Panjabi Sumeet S., Dimopoulos Meletios (2019). Convenience, satisfaction, health-related quality of life of once-weekly 70 mg/m2 vs. twice-weekly 27 mg/m2 carfilzomib (randomized A.R.R.O.W. study). Leukemia.

[14] Siegel DS (2012). A phase 2 study of single-agent carfilzomib (PX-171-003-A1) in patients with relapsed and refractory multiple myeloma. Blood.

[15] KYPROLIS®. *KYPROLIS® (carfilzomib) [prescribing information]* (Onyx Pharmaceuticals, Inc, South San Francisco, CA, 2018).

[16] Dimopoulos MA (2016). Carfilzomib and dexamethasone versus bortezomib and dexamethasone for patients with relapsed or refractory multiple myeloma (ENDEAVOR): a randomised, phase 3, open-label, multicentre study. Lancet Oncol..

[17] Stewart AK (2015). Carfilzomib, lenalidomide, and dexamethasone for relapsed multiple myeloma. N. Engl. J. Med..

[18] Siegel DS (2018). Improvement in overall survival with carfilzomib, lenalidomide, and dexamethasone in patients with relapsed or refractory multiple myeloma. J. Clin. Oncol..

[19] Dimopoulos MA (2017). Carfilzomib or bortezomib in relapsed or refractory multiple myeloma (ENDEAVOR): an interim overall survival analysis of an open-label, randomised, phase 3 trial. Lancet Oncol..

[20] Dimopoulos MA (2017). Carfilzomib, lenalidomide, and dexamethasone in patients with relapsed multiple myeloma categorised by age: secondary analysis from the phase 3 ASPIRE study. Br. J. Haematol..

[21] Ludwig H (2017). Carfilzomib and dexamethasone vs bortezomib and dexamethasone in patients with relapsed multiple myeloma: results of the phase 3 study ENDEAVOR (NCT01568866) according to age subgroup. Leuk. Lymphoma.

[22] Dimopoulos MA (2017). Carfilzomib-lenalidomide-dexamethasone vs lenalidomide-dexamethasone in relapsed multiple myeloma by previous treatment. Blood Cancer J..

[23] Moreau P (2017). Impact of prior treatment on patients with relapsed multiple myeloma treated with carfilzomib and dexamethasone vs bortezomib and dexamethasone in the phase 3 ENDEAVOR study. Leukemia.

[24] Avet-Loiseau H (2016). Carfilzomib significantly improves the progression-free survival of high-risk patients in multiple myeloma. Blood.

[25] Chng WJ (2017). Carfilzomib-dexamethasone vs bortezomib-dexamethasone in relapsed or refractory multiple myeloma by cytogenetic risk in the phase 3 study ENDEAVOR. Leukemia.

[26] Dimopoulos MA (2017). Superior efficacy of carfilzomib and dexamethasone (Kd56) vs bortezomib and dexamethasone (Vd) in multiple myeloma (MM) patients with moderate or serious renal failure: a subgroup analysis of the phase 3 ENDEAVOR study. Blood.

[27] Moreau P (2018). Once weekly versus twice weekly carfilzomib dosing in patients with relapsed and refractory multiple myeloma (A.R.R.O.W.): interim analysis results of a randomised, phase 3 study. Lancet Oncol..

[28] Durie BG (2006). International uniform response criteria for multiple myeloma. Leukemia.

[29] Rajkumar SV (2011). Consensus recommendations for the uniform reporting of clinical trials: report of the International Myeloma Workshop Consensus Panel 1. Blood.

[30] Repetto L (2003). Greater risks of chemotherapy toxicity in elderly patients with cancer. J. Support. Oncol..

[31] Yancik R, Ganz PA, Varricchio CG, Conley B (2001). Perspectives on comorbidity and cancer in older patients: approaches to expand the knowledge base. J. Clin. Oncol..

[32] Qian Y (2017). Renal impairment and use of nephrotoxic agents in patients with multiple myeloma in the clinical practice setting in the United States. Cancer Med..

[33] Gabardi S, Abramson S (2005). Drug dosing in chronic kidney disease. Med. Clin. North Am..

[34] Kurtin SE (2013). Relapsed or relapsed/refractory multiple myeloma. J. Adv. Pract. Oncol..

[35] Ziogas DC, Terpos E, Kastritis E, Dimopoulos MA (2017). An overview of the role of carfilzomib in the treatment of multiple myeloma. Expert Opin. Pharmacother..

[36] Muchtar E (2016). Efficacy and safety of salvage therapy using Carfilzomib for relapsed or refractory multiple myeloma patients: a multicentre retrospective observational study. Br. J. Haematol..

[37] Facon T (2018). Final analysis of survival outcomes in the phase 3 FIRST trial of up-front treatment for multiple myeloma. Blood.

[38] Pulte ED (2018). FDA approval summary: lenalidomide as maintenance therapy after autologous stem cell transplant in newly diagnosed multiple myeloma. Oncologist.

[39] Dimopoulos MA, Terpos E, Niesvizky R, Palumbo A (2015). Clinical characteristics of patients with relapsed multiple myeloma. Cancer Treat. Rev..

[40] Palumbo A (2014). International Myeloma Working Group consensus statement for the management, treatment, and supportive care of patients with myeloma not eligible for standard autologous stem-cell transplantation. J. Clin. Oncol..

[41] National Comprehensive Cancer Network (NCCN). NCCN Clinical practice guidelines in oncology: multiple myeloma version 2.2019. https://www.nccn.org/professionals/physician_gls/pdf/myeloma.pdf (2019). Accessed 15 October (2019).

[42] Mikhael J (2019). Treatment of multiple myeloma: ASCO and CCO Joint Clinical Practice Guideline. J. Clin. Oncol..

[43] Dimopoulos MA (2016). Daratumumab, lenalidomide, and dexamethasone for multiple myeloma. N. Engl. J. Med..

[44] Dimopoulos MA (2018). Daratumumab plus lenalidomide and dexamethasone versus lenalidomide and dexamethasone in relapsed or refractory multiple myeloma: updated analysis of POLLUX. Haematologica.

[45] Palumbo A (2016). Daratumumab, bortezomib, and dexamethasone for multiple myeloma. N. Engl. J. Med..

[46] Spencer A (2018). Daratumumab plus bortezomib and dexamethasone versus bortezomib and dexamethasone in relapsed or refractory multiple myeloma: updated analysis of CASTOR. Haematologica.

[47] Mateos Maria-Victoria, Spencer Andrew, Nooka Ajay K., Pour Ludek, Weisel Katja, Cavo Michele, Laubach Jacob P., Cook Gordon, Iida Shinsuke, Benboubker Lotfi, Usmani Saad Z., Yoon Sung-Soo, Bahlis Nizar J., Chiu Christopher, Ukropec Jon, Schecter Jordan M., Qin Xiang, O’Rourke Lisa, Dimopoulos Meletios A. (2019). Daratumumab-based regimens are highly effective and well tolerated in relapsed or refractory multiple myeloma regardless of patient age: subgroup analysis of the phase 3 CASTOR and POLLUX studies. Haematologica.

