# Supplementary material for: Once- versus twice-weekly carfilzomib in relapsed and refractory multiple myeloma by select patient characteristics: phase 3 A.R.R.O.W. study subgroup analysis
Source: Blood Cancer J. 2020 Mar 9;10(3):35. doi: 10.1038/s41408-020-0300-y (PMC7062899; doi:10.1038/s41408-020-0300-y)
Supplement: Supplementary file 1 — Supplemental Material [file 41408_2020_300_MOESM1_ESM.doc]

**Supplementary Information**

**Title: Once- vs twice-weekly carfilzomib in relapsed and refractory multiple myeloma by select patient characteristics: phase 3 ARROW study subgroup analysis**

Authors: Meletios A Dimopoulos^1^, Ruben Niesvizky^2^, Katja Weisel^3^, David S Siegel^4^, Roman Hajek^5^, María-Victoria Mateos^6^, Michele Cavo^7^, Mei Huang^8^, Anita Zahlten-Kumeli^8^, and Philippe Moreau^9^

Supplemental Table S1. Select baseline demographics with ≥10% difference across treatment arms by age subgroups

| **Baseline characteristic** | <65 years | | 65–74 years | | ≥75 years | |
| --- | --- | --- | --- | --- | --- | --- |
|  | Once-weekly Kd70 mg/m^2^  (n=104) | Twice-weekly Kd27 mg/m^2^  (n=104) | Once-weekly Kd70 mg/m^2^  (n=90) | Twice-weekly Kd27 mg/m^2^  (n=102) | Once-weekly Kd70 mg/m^2^  (n=46) | Twice-weekly Kd27 mg/m^2^  (n=32) |
| **Geographic region** |  |  |  |  |  |  |
| Asia Pacific | – | – | 16 (17.8) | 6 (5.9) | – | – |
| **ECOG PS** |  |  |  |  |  |  |
| 0 | – | – | – | – | 19 (41.3) | 9 (28.1) |
| 1 | – | – | – | – | 27 (58.7) | 23 (71.9) |
| **Baseline CrCL** |  |  |  |  |  |  |
| 30 to <50 mL/min | – | – | – | – | 26 (56.5) | 13 (40.6) |
| 50 to <80 mL/min | – | – | 49 (54.4) | 67 (65.7) | – | – |
| **Risk group as determined by FISH^a^** |  |  |  |  |  |  |
| High | – | – | 7 (7.8) | 20 (19.6) | – | – |
| Standard | – | – | – | – | 6 (13.0) | 11 (34.4) |
| Unknown | – | – | – | – | 36 (78.3) | 20 (62.5) |
| **Prior lines of therapy** |  |  |  |  |  |  |
| 2 | – | – | – | – | 17 (37.0) | 19 (59.4) |
| 3 | – | – | – | – | 29 (63.0) | 13 (40.6) |
| **Received bortezomib-including regimen** |  |  |  |  |  |  |
| Yes | – | – | – | – | – | – |
| Refractory to any prior bortezomib-  including regimen | – | – | 43 (47.8) | 33 (32.4) | 25 (54.3) | 21 (65.6) |
| Not refractory to any prior bortezomib-  including regimen | – | – | 47 (52.2) | 68 (66.7) | – | – |
| **Received lenalidomide-including regimen** |  |  |  |  |  |  |
| Yes | – | – | – | – | 44 (95.7) | 22 (68.8) |
| Refractory to any prior lenalidomide-  including regimen | – | – | – | – | 41 (89.1) | 18 (56.3) |
| No | – | – | – | – | 2 (4.3) | 10 (31.3) |
| Values reported as n (%).  Dashes (–) correspond to differences <10% between treatment arms.  Select baseline characteristics differing by ≥10% between treatment arms are limited to characteristics presented in Moreau et al. 2018 Lancet Oncol.  ^a^Genetic abnormalities t(4; 14), t(14;16), and del(17p) are in the high risk group, the others are in the standard risk group.  CrCL, creatinine clearance; ECOG PS, Eastern Cooperative Oncology Group performance status; FISH, fluorescence in situ hybridization; ISS, International Staging System. | | | | | | |

Supplemental Table S2. Select baseline demographics with ≥10% difference across treatment arms by baseline renal function

| **Baseline characteristic** | CrCL <50 mL/min | | CrCL 50 to <80 mL/min | | CrCL ≥80 mL/min | |
| --- | --- | --- | --- | --- | --- | --- |
|  | Once-weekly Kd70 mg/m^2^  (n=50) | Twice-weekly Kd27 mg/m^2^  (n=35) | Once-weekly Kd70 mg/m^2^  (n=91) | Twice-weekly Kd27 mg/m^2^  (n=111) | Once-weekly Kd70 mg/m^2^  (n=99) | Twice-weekly Kd27 mg/m^2^  (n=91) |
| **Age** |  |  |  |  |  |  |
| 18 – 64 years | 6 (12.0) | 9 (25.7) | – | – | – | – |
| 75 – 84 years | 26 (52.0) | 14 (40.0) | – | – | – | – |
| **Geographic region** |  |  |  |  |  |  |
| Asia Pacific | – | – | 17 (18.7) | 8 (7.2) | – | – |
| Europe | 42 (84.0) | 33 (94.3) | – | – | – | – |
| **Risk group as determined by FISH^a^** |  |  |  |  |  |  |
| Unknown | – | – | 61 (67.0) | 62 (55.9) | – | – |
| **ISS stage at baseline^b^** |  |  |  |  |  |  |
| Stage 1 and 2 | 22 (44.0) | 19 (54.3) | – | – | – | – |
| **Previous transplant** |  |  |  |  |  |  |
| Yes | – | – | 44 (48.4) | 65 (58.6) | – | – |
| No | – | – | 47 (51.6) | 46 (41.4) | – | – |
| **Prior lines of therapy** |  |  |  |  |  |  |
| 2 | – | – | 39 (42.9) | 59 (53.2) | – | – |
| 3 | – | – | 52 (57.1) | 52 (46.8) | – | – |
| **Received bortezomib-including regimen** |  |  |  |  |  |  |
| Yes | – | – | – | – | – | – |
| Refractory to any prior bortezomib-  including regimen | – | – | – | – | 38 (38.4) | 25 (27.5) |
| Not refractory to any prior bortezomib-  including regimen | – | – | – | – | 59 (59.6) | 66 (72.5) |
| **Received lenalidomide-including regimen** |  |  |  |  |  |  |
| Yes | 47 (94.0) | 23 (65.7) | – | – | – | – |
| Refractory to any prior lenalidomide-  including regimen | 39 (78.0) | 21 (60.0) | 76 (83.5) | 79 (71.2) | – | – |
| Not refractory to any prior lenalidomide-  including regimen | 8 (16.0) | 2 (5.7) | – | – | – | – |
| No | 3 (6.0) | 12 (34.3) | – | – | – | – |
| **Received thalidomide-including regimen** |  |  |  |  |  |  |
| Yes | 17 (34.0) | 23 (65.7) | – | – | – | – |
| Refractory to any prior thalidomide-  including regimen | 7 (14.0) | 12 (34.3) | – | – | – | – |
| Not refractory to any prior thalidomide-  including regimen | 10 (20.0) | 11 (31.4) | – | – | – | – |
| No | 33 (66.0) | 12 (34.3) | – | – | – | – |
| Values reported as n (%).  Dashes (–) correspond to differences <10% between treatment arms.  Select baseline characteristics differing by ≥10% between treatment arms are limited to characteristics presented in Moreau et al. 2018 Lancet Oncol.  ^a^Genetic abnormalities t(4; 14), t(14;16), and del(17p) are in the high risk group, the others are in the standard risk group.  ^b^Sponsor-derived.  ^c^Exact ISS stage could not be decided for some subjects in this group because of missing beta-2 microglobulin.  CrCL, creatinine clearance; FISH, fluorescence in situ hybridization; ISS, International Staging System. | | | | | | |

Supplemental Table S3. Select baseline demographics with ≥10% difference across treatment arms by prior lines of therapy

| **Baseline characteristic** | 2 prior lines | | 3 prior lines | |
| --- | --- | --- | --- | --- |
|  | Once-weekly Kd70 mg/m^2^  (n=116) | Twice-weekly Kd27 mg/m^2^  (n=125) | Once-weekly Kd70 mg/m^2^  (n=124) | Twice-weekly Kd27 mg/m^2^  (n=113) |
| **Age** |  |  |  |  |
| 75–84 years | – | – | 28 (22.6) | 13 (11.5) |
| **Baseline CrCL** |  |  |  |  |
| 50 to <80 mL/min | 39 (33.6) | 59 (47.2) | – | – |
| **Risk group as determined by FISH^a^** |  |  |  |  |
| Standard risk | 19 (16.4) | 37 (29.6) | – | – |
| Unknown | 76 (65.5) | 63 (50.4) | – | – |
| **Prior transplant** |  |  |  |  |
| Yes | – | – | 70 (56.5) | 77 (68.1) |
| Autologous | – | – | 69 (55.6) | 76 (67.3) |
| No | – | – | 54 (43.5) | 36 (31.9) |
| **Received bortezomib-including regimen** |  |  |  |  |
| Yes | – | – | – | – |
| Refractory to any prior bortezomib-including regimen | – | – | 66 (53.2) | 47 (41.6) |
| Not refractory to any prior bortezomib-including regimen | – | – | 56 (45.2) | 66 (58.4) |
| **Received lenalidomide-including regimen** |  |  |  |  |
| Yes | – | – | – | – |
| Refractory to any prior lenalidomide-including regimen | 85 (73.3) | 79 (63.2) | – | – |
| Values reported as n (%).  Dashes (–) correspond to differences <10% between treatment arms.  Select baseline characteristics differing by ≥10% between treatment arms are limited to characteristics presented in Moreau et al. 2018 Lancet Oncol.  ^a^Genetic abnormalities t(4; 14), t(14;16), and del(17p) are in the high risk group, the others are in the standard risk group.  CrCL, creatinine clearance; FISH, fluorescence in situ hybridization; ISS, International Staging System. | | | | |

Supplemental Table S4. Select baseline demographics with ≥10% difference across treatment arms by bortezomib refractory status

| **Baseline characteristic** | Refractory to bortezomib | | Not refractory to bortezomib | |
| --- | --- | --- | --- | --- |
|  | Once-weekly Kd70 mg/m^2^  (n=111) | Twice-weekly Kd27 mg/m^2^  (n=90) | Once-weekly Kd70 mg/m^2^  (n=129) | Twice-weekly Kd27 mg/m^2^  (n=148) |
| **Age** |  |  |  |  |
| 65 – 74 years | – | – | 47 (36.4) | 69 (46.6) |
| **Risk group as determined by FISH^a^** |  |  |  |  |
| Unknown/missing | – | – | 88 (68.2) | 84 (56.8) |
| **Baseline CrCL** |  |  |  |  |
| 50 to <80 mL/min | 43 (38.7) | 44 (48.9) | – | – |
| **Received lenalidomide-including regimen** |  |  |  |  |
| Yes | 92 (82.9) | 63 (70.0) | – | – |
| Refractory to any prior lenalidomide-including regimen | 79 (71.2) | 48 (53.3) | – | – |
| No | 19 (17.1) | 27 (30.0) | – | – |
| Values reported as n (%).  Dashes (–) correspond to differences <10% between treatment arms.  Select baseline characteristics differing by ≥10% between treatment arms are limited to characteristics presented in Moreau et al. 2018 Lancet Oncol.  ^a^Genetic abnormalities t(4; 14), t(14;16), and del(17p) are in the high risk group, the others are in the standard risk group.  CrCL, creatinine clearance; FISH, fluorescence in situ hybridization. | | | | |

Supplemental Table S5. Treatment-emergent grade ≥3 adverse events of interest by ECOG PS and ISS stage

| **Subgroup** | **ECOG PS: 0** | | **ECOG PS: 1** | | **ISS: 1 and 2** | | **ISS: 3** | |
| --- | --- | --- | --- | --- | --- | --- | --- | --- |
|  | **Once-weekly Kd70 mg/m^2^**  (n = 117) | **Twice-weekly Kd27 mg/m^2^**  (n = 116) | **Once-weekly Kd70 mg/m^2^**  (n = 120) | **Twice-weekly Kd27 mg/m^2^**  (n = 119) | **Once-weekly Kd70 mg/m^2^**  (n = 173) | **Twice-weekly Kd27 mg/m^2^**  (n = 177) | **Once-weekly Kd70 mg/m^2^**  (n = 62) | **Twice-weekly Kd27 mg/m^2^**  (n = 54) |
| Peripheral neuropathy | 0 | 1 (0.9) | 0 | 0 | 0 | 1 (0.6) | 0 | 0 |
| Acute renal failure | 4 (3.4) | 5 (4.3) | 5 (4.2) | 8 (6.7) | 3 (1.7) | 7 (4.0) | 6 (9.7) | 6 (11.1) |
| Acute kidney injury | 4 (3.4) | 3 (2.6) | 4 (3.3) | 5 (4.2) | 3 (1.7) | 6 (3.4) | 5 (8.1) | 2 (3.7) |
| Cardiac failure | 2 (1.7) | 3 (2.6) | 5 (4.2) | 7 (5.9) | 4 (2.3) | 7 (4.0) | 3 (4.8) | 2 (3.7) |
| Ischemic heart disease | 1 (0.9) | 1 (0.9) | 1 (0.8) | 1 (0.8) | 2 (1.2) | 0 | 0 | 2 (3.7) |
| Pulmonary hypertension | 0 | 0 | 0 | 1 (0.8) | 0 | 0 | 0 | 1 (1.9) |
| Hypertension | 7 (6.0) | 6 (5.2) | 7 (5.8) | 7 (5.9) | 11 (6.4) | 11 (6.2) | 3 (4.8) | 2 (3.7) |
| Anemia | 14 (12.0) | 22 (19.0) | 28 (23.3) | 20 (16.8) | 22 (12.7) | 26 (14.7) | 20 (32.3) | 15 (27.8) |
| Thrombocytopenia | 8 (6.8) | 8 (6.9) | 9 (7.5) | 8 (6.7) | 10 (5.8) | 10 (5.6) | 7 (11.3) | 6 (11.1) |
| Neutropenia | 7 (6.0) | 11 (9.5) | 7 (5.8) | 5 (4.2) | 11 (6.4) | 8 (4.5) | 3 (4.8) | 8 (14.8) |
| Cardiac arrhythmias | 1 (0.9) | 1 (0.9) | 1 (0.8) | 2 (1.7) | 0 | 3 (1.7) | 2 (3.2) | 0 |
| Myocardial infarction | 0 | 1 (0.9) | 1 (0.8) | 0 | 1 (0.6) | 0 | 0 | 1 (1.9) |
| All values are n (%).  TEAEs are defined as any adverse event with an onset date from the first dose through 30 days after the last dose of any study drug.  Adverse events were coded using MedDRA version 20.0 and graded using NCI-CTCAE (version 4.03).  Subjects were counted only once for each search strategy and each preferred term.  Adverse events (peripheral neuropathy, cardiac failure, ischemic heart disease, pulmonary hypertension, cardiac arrhythmias, and myocardial infarction) are listed as SMQ, narrow scope or preferred terms (acute kidney injury, anemia, thrombocytopenia, and neutropenia).  ECOG PS, Eastern Cooperative Oncology Group performance status; ISS, International Staging System; MedDRA, Medical Dictionary for Regulatory Activities; NCI-CTCAE, National Cancer Institute-Common Terminology Criteria for Adverse Events; SMQ, Standardized MedDRA Queries; TEAE, treatment-emergent adverse event. | | | | | | | | |
